# The impact of health shocks on housing instability: Evidence from urban Medicaid enrollees

**DOI:** 10.1016/j.jhealeco.2026.103150

**Published:** 2026-05-19

**Authors:** Kacie L. Dragan

**Affiliations:** a Columbia University Mailman School of Public Health, Department of Health Policy and Management, 722 West 168th St., 4th Floor, NY, NY 10032, United States; b Harvard University, Graduate School of Arts and Sciences, Interfaculty Initiative in Health Policy, 8 Story St. Suite 380, Cambridge, MA 02138, United States

**Keywords:** Medicaid, Social Policy, Housing, Hospitalizations, Health, Urban economics, Homelessness

## Abstract

Poor health and unstable housing are closely linked. Most research has focused on how housing shapes health, with little empirical study of whether and how health events can lead to future residential mobility or housing instability. This paper uses high-frequency administrative data on residential location and health among Medicaid enrollees in New York City to test whether adverse health events trigger housing mobility or insecurity, independent of the financial toll of medical bills. Using an event study design, I find that health shocks – or, sudden hospitalizations after two hospital-free years – immediately increase residential mobility (21–35 % relative increase) and the probability of living in shelters or on the street (6–10 % relative increase). These increased rates of mobility and instability persist above expected levels for at least two years. For unplanned or urgent hospital admissions, the impact of health events is even greater. These estimates imply that, in their immediate aftermath, adverse health events could be a tipping point for approximately 80,000 additional moves and 20,000 additional cases of homelessness among the U.S. Medicaid-insured population annually. The effects of health events on residential mobility are smaller for those with subsidized housing, a usual source of outpatient care, higher-quality inpatient care, and social support, suggesting potential areas for policy interventions to break the relationship between health problems and housing outcomes, from both inside and outside of health systems. This work also contributes to our understanding of the long tail of social consequences of adverse health events.

## 1. Introduction

Housing and health are closely linked: people experiencing residential instability or inadequate housing have been found to have higher rates of a range of mental and physical health conditions (Collinson et al., 2024; Meltzer and Schwartz, 2016; Pollack et al., 2010; Routhier et al., 2023; Swope and Hernández, 2019). The relationship between the two is widely speculated to be bidirectional and cyclical, and health systems and insurers have begun to take an interest in using health sector interventions to help precariously-housed patients. Yet, the bulk of research has only focused on estimating how housing is a determinant of health status. In contrast, little attention has been paid to the role of health events themselves in shaping housing outcomes, in which a person’s *housing* circumstances may be a consequence of adverse *health* events.

This paper uses high-frequency housing and health data in event study analyses to ask whether health events cause housing mobility or insecurity – and what features of health systems or housing systems may protect low-income people from this phenomenon. Understanding the causes of mobility and housing insecurity is a significant policy priority. In recent years, rates of homelessness have been increasing, with up to 1.25 million Americans annually experiencing homelessness – or the state of sleeping in a shelter or on the street for at least one night (United States Interagency Council on Homelessness, 2022). Many more face other forms of housing instability, including the 60–80 % of low-income Americans considered “housing cost burdened” and the one million families in hotels, motels, or doubled-up with relatives (Mateyka and Yoo, 2023; Office of Community Planning and Development, 2023). These housing challenges occur against the backdrop of increasing rates of long-term chronic diseases, as well as growing attention by health systems to patients’ social needs (Boersma et al., 2020; Horwitz et al., 2020).

Housing costs – and scarcity of affordable housing – have been theorized as core drivers of residential instability in the United States (Colburn and Aldern, 2022; Lee et al., 2003; O’Flaherty, 2019, 2011; Quigley and Raphael, 2001; Shinn and Khadduri, 2020). Yet, conditional on harsh housing markets, both theoretical and empirical literature suggest that there are a host of other causes that may act as key tipping points (Meyer et al., 2024; O’Flaherty, 2009). One potential form of insulation against falling into housing insecurity is a person’s health. Whether due to poor access or quality of care, social factors, or mere bad luck, health problems may kick off a broader downward spiral in wellbeing (Cutler et al., 2014; García-Gómez et al., 2013; Mohanan, 2013). As a result, other domains of life – including securing or maintaining employment, social support, or stable housing – become challenging (National Health Care for the Homeless Council, 2019). In interview-based studies, up to half of people experiencing residential instability cite health problems as a contributor, often above and beyond the direct financial toll of medical bills (Doran et al., 2019; Jarwala and Singh, 2019; Kushel et al., 2023). Yet, prior work suggests that Americans are incompletely insured against the effects of health events, in both formal or informal forms of insurance (Dobkin et al., 2018; O’Flaherty, 2011, 2009).

This paper empirically tests whether sudden health events act as tipping points into future housing mobility and insecurity, for whom this risk is greatest, and factors within health systems and other social service sectors that might be protective against this relationship. I use novel high-frequency data that links residential move and address information to health information for Medicaid enrollees in New York City (NYC). I use a quasi-experimental event study approach to test whether there is increase in markers of residential mobility and homelessness immediately after a “health shock,” defined as a sudden hospitalization following two hospital-free years, in line with prior health shocks research (Dobkin et al., 2018). I then test for heterogeneity in the size of this increase across policy-relevant dimensions, including access to subsidized housing, quality of health care, and social support.

This approach has several strengths for answering the question of whether adverse health events can precipitate housing instability. First, prior research has largely relied on survey data with multi-year gaps between survey waves, making it difficult to separate the timing of adverse health events from adverse housing events. The real-time frequency of residential and health information in this paper overcomes this challenge by measuring exact timing of moves relative to a well-defined, sudden adverse health event. Additionally, this research uses data from NYC, which has high baseline rates of housing instability, making this a salient setting with a large enough population to detect relationships. Supplemental data from NYC agencies is also very rich, including the ability to link to characteristics like building quality, hospital quality, family composition, and subsidized housing status to understand effect heterogeneity.

Finally, using data from a state with virtually no Medicaid cost-sharing allows for the isolation of the role of health itself on housing outcomes, separate from the direct financial impact of medical bills on a person’s ability to afford housing (Ali et al., 2024; Allen et al., 2019; Dobkin et al., 2018). Understanding how health events make people uniquely vulnerable to mobility and housing instability –beyond the impact of medical costs – is important, as many low-income people are already on zero-cost-sharing plans (or nearly so).

In this paper, I find that health shocks lead to an immediate increase in residential mobility and instability. Specifically, health shocks generate an increase of 10.1 to 19.0 additional moves per 1000 enrollees per quarter (a 21–35 % relative increase, from a baseline rate of about 50) and a 0.1 to 0.3 percentage point increase in extreme mobility, or the share moving two or more times per quarter (a 40–56 % relative increase, from a baseline rate of about 0.4 percent). Mobility rates persist above expected levels for at least two years following the health event. I observe smaller, though still significant, effects on the probability of living in particularly unstable situations (e.g., in shelters, on the street), at a 6–10 % increase. The magnitude of the increases is smaller for those with access to subsidized housing, a usual source of outpatient care, social support, and higher quality inpatient care. This heterogeneity suggests that there may be opportunities to intervene in the relationship between health problems and housing instability, from both within and outside of health systems.

These findings are robust to a wide range of sensitivity analyses and tests of the underlying assumptions, strengthening the interpretation that these estimates isolate the genuine role of health events. While other unobservable major life events, like divorce or job loss, may plausibly precede an adverse health event and also cause housing instability, I find no evidence that family dissolution rates increase before or at the time of the health shock, nor do I find sizeable changes in probability of continuous Medicaid enrollment, which might indicate labor market events. I observe similar magnitudes regardless of whether a child or adult experiences the health shock. This supports the notion that the health event may be the primary force driving changes in housing outcomes: child health shocks are less likely to be related to confounding events like divorce or job loss, and observing similarly strong effects on housing outcomes when a *child* in the home experiences a health event supports the assumption that the health events identified by this strategy are not just proxies for other life events. Finally, I find compelling evidence that these address-based moves do not merely reflect higher likelihood of administrative address updates that are generated when enrollees engage with the Medicaid program and their providers during a period of illness: there is no meaningful increase in moves when enrollees experience non-urgent admissions, like joint replacements or bariatric surgeries. Additionally, I can replicate the main results even when using an alternative definition of housing instability – the presence of an ICD-10 diagnostic code for “problems with housing” – which does not rely on administrative addresses at all.

When considered in the broader US context, the estimates from this paper imply two core messages relevant to housing and health policy. First, health events, in their immediate wake, may act as the tipping point for about 80,000 instances of residential mobility and 20,000 cases of homelessness among low-income Medicaid enrollees in the US annually. The relative increases in housing instability are similar in magnitude to those estimated in the literature on other life shocks, such as a felony conviction, divorce, and job loss. Second, about 40 % of the naïve, cross-sectional association between hospitalization rates and residential mobility typically observed in low-income populations may actually be attributable to *the role of health events on housing outcomes* (rather than the reverse, or due to common third factors). This is a key insight, as most prior work has interpreted the correlation between poor health and housing status as *only* reflecting housing as a social determinant of health – not also housing mobility as a consequence of health events.

These findings also fill several gaps in the housing-health literature and in our understanding of the challenges low-income families face. First, this paper contributes to our understanding of the complex bidirectional relationship between housing and health. Specifically, it acts as a complement to the body of work that has aimed to quantify the role of housing as a social determinant of health by using a quasi-experimental approach to examine the relationship in the opposite direction. Good health may be challenging to achieve when a person is facing housing insecurity, and these results show that stable housing may likewise be challenging to maintain when a person is facing a health crisis. While this analytic strategy cannot comment on the *relative contribution* of health events to housing instability versus other component causes (like housing market fluctuations), it does cleanly document an immediate, substantial increase in mobility and instability after a health shock. Second, these findings also add to our understanding of the long tail of social and economic consequences of serious health events on people’s lives. Stephens Jr. and Toohey (2022), Dobkin et al. (2018), and García-Gómez et al. (2013) have documented the financial and labor market impacts of health events. This paper adds to this literature by showing that health shocks, even independent of any direct financial impact of medical bills, may disrupt housing outcomes as well. This reveals a new dimension of the tightly entwined housing-health relationship.

## 2. Background and theory

### 2.1. The bidirectional relationship between housing and health

A large literature has investigated housing as a determinant of health. Unaffordable housing has been linked to challenges paying for health-promoting goods; low-quality housing has been associated with exposure to hazards like mold or pests which may harm physical health; and unstable housing or forced moves have been associated with increased stress and acute care use (Collinson et al., 2024; Meltzer and Schwartz, 2016; Pollack et al., 2010; Routhier et al., 2023; Swope and Herńandez, 2019). The smaller body of work studying reverse pathways between social factors and health, however, has focused on the relationship between health and financial outcomes, rather than housing outcomes (Cutler et al., 2011; García-Gómez et al., 2013; Mohanan, 2013; Stephens Jr. and Toohey, 2022). Of particular note, Dobkin et al. (2018) documented the impact of health events on earnings, medical debt, and bankruptcy. Similarly, research in Scandinavia found long-lasting impacts to parental earnings following a health shock to one’s child (Breivik and Costa-Ramón, 2021; Eriksen et al., 2021). These findings demonstrate that health crises have broader-scale impacts beyond clinical outcomes.

Most glimpses of the phenomenon of health selection into adverse housing situations have come from studies examining housing as a social determinant of health, which have incidentally noted a “run-up” of health problems before a housing problem as a peripheral, descriptive finding (Collinson et al., 2024; Okuzono et al., 2023; Treglia et al., 2019). At the neighborhood level, Arcaya et al. (2016) tested for health selection into adverse residential circumstances by re-analyzing data from the experimental Moving to Opportunity study, finding families with sick children were unlikely to opt to move to a better neighborhood. Alongside similar results from observational data (Rolheiser et al., 2022), this suggests poor health may constrain exits from residential poverty or induce moves into high-poverty areas.

The small literature supporting health events as a cause of adverse housing outcomes largely relies on survey data with infrequent waves, making it difficult to comment on temporality between the events.^1^ The work best able to parse out temporality at the individual level exclusively focuses on births. Curtis et al. (2013), Schwartz et al. (2021), and Gold and Wagner (2022) used the Fragile Families survey to leverage births of children with congenital (or other) health problems as a plausibly random shock, finding they were more likely to experience evictions and homelessness. In county-level analyses, Bradford and Maclean (2024) noted an association between opening of new psychiatric centers and reductions in local eviction rates, as well as a relationship between the rollout of pharmaceutical policies that fueled the opioid crisis and adverse housing outcomes (Bradford et al., 2024). Health care delivery and health policy may play a key role in housing stability.

However, no studies have used high-frequency individual-level data in a quasi-experimental framework to clearly establish temporality and estimate causal effects of health events on housing outcomes across a range of conditions or among a diverse and broad population. Further, most research has been limited to measuring only what is known as “literal homelessness” – or the state of sleeping on the street or in a shelter – or to measuring only formal evictions. Yet, the increasing rates of other forms of housing mobility and instability, like couch surfing and “doubling up”, informal (coercive) evictions,^2^ and short-term arrangements like motels and hostels, increase the urgency around understanding the role health events can play for residential mobility more generally. Finally, existing literature has been unable to isolate the role of the health event itself separate from the financial impact of medical bills – an important distinction, as many low-income people are insured by Medicaid, which has little to no cost-sharing for enrollees.

### 2.2. Pathways between a health event and subsequent housing instability

One common model for conceptualizing the determinants of housing instability posits that Americans are poorly insured against life shocks (such as income shocks, relationship shocks, and health shocks). This generates a world in which landlords may become an “insurer of last resort” when families are unable to make rent or satisfy other lease terms, increasing risk of formal or informal evictions and the restrictive screening of tenants preemptively (Desmond and Gershenson, 2017; O’Flaherty, 2011). Combined with inadequate supply of housing – which is believed to fuel high rents in many US cities (Quigley and Raphael, 2001) – life shocks may tip people on the precipice into housing instability. Health events are one particular life shock against which people are poorly insured (Dobkin et al., 2018), both formally (inadequate health insurance or social insurance) and informally (inadequate social support or quality of care for low-income patients). Building on the empirical literature on drivers of housing instability (Bryan, 2023; Lee et al., 2003; Meyer et al., 2024; O’Flaherty, 2019, 2009) and qualitative research on the experiences of people facing housing instability (Desmond, 2016; Doran et al., 2019; Jarwala and Singh, 2019; Shinn and Khadduri, 2020), this paper explores three broad mechanisms that might drive larger or smaller relationships between a health shock and subsequent risk of housing instability, outside the direct burden of medical bills.

First, income or the ability to pay rent may be disrupted, generating informal or formal evictions, landlord or roommate disputes, or desires to find cheaper housing. Policies that increase insurance – thus reducing out-of-pocket costs – have been shown to reduce eviction rates and related financial outcomes like bankruptcy (Ali et al., 2024; Allen et al., 2019; Hu et al., 2018). However, little is known about whether health events, independent of the toll of medical expenses, matter for instability. People who fall ill are known to be more likely to lose their jobs, see reduced wages, or be unable to return to work (Dobkin et al., 2018; García-Gómez et al., 2013; Smalligan and Mudrazija, 2019), and qualitative literature has documented that people can have trouble paying rent or meeting lease conditions (Doran et al., 2019; Jarwala and Singh, 2019; Shinn and Khadduri, 2020), making this a plausible pathway.

Second, assets like access to subsidized housing, a robust social support network, and usual sources of health care may boost resilience as “implicit insurance,” whereby the impact of disruptive health events is blunted by pre-existing supports enrollees can draw on. Health shocks have been shown to strain caregiver support networks (Davidson et al., 2008; Gérain and Zech, 2019; Wittenberg and Prosser, 2013) and the risks of social isolation for recovery during health crises have been explored in a number of settings (e.g., in Klinenberg’s (2015) “autopsy” of the Chicago Heat Wave). Recent county-level analyses have documented a link between the opening of new psychiatric treatment centers and reduced area-level eviction rates, further supporting the plausibility of this mechanism (Bradford and Maclean, 2024). The presence of – and access to – supportive institutions or networks may offer protection in the face of adversity, like a psychiatric crisis or unexpected injury. Those whose rent makes up a smaller share of their income, those who live with family who can “pick up the slack”, or those who have strong pre-existing relationships with their health providers may be buoyed during health crises in ways that others are not.

Lastly, the adequacy of safety net programs designed to “catch” or rehabilitate people in crisis may generate variation. In theory, the safety net should be most agile and responsive precisely in times of crisis. But inadequate, inefficient, or inaccessible social services may inhibit recovery. Hospital quality has been shown to be a key factor in recovery trajectories, including generating longer lengths of stay, complications, and re-admissions (Krumholz et al., 2017), which could destabilize people’s lives more permanently. Likewise, policies in the housing sector vary in flexibility, which can have consequences for tenants in recovery (Preston and Reina, 2021): if payments can’t be adjusted in response to income (as they can in public housing), the risk of eviction during a health crisis may be greater. Insufficient protections against discrimination by landlords is also a potential concern, as audit studies have shown that landlords are less likely to rent to or make accommodations for people with disabilities (e.g., wheelchair, hearing assistance) or mental health conditions (Hammel et al., 2017; Levy et al., 2015).

## 3. Empirical approach

### 3.1. Data and sample

The data used in this analysis include NYC Medicaid claims, encounters, and demographic information from 2010–2019. I used all enrollees with an inpatient stay between 2012 and 2017, following a 2-year period of being hospitalization-free. The “exposure” is thus the exact date of sudden hospitalization. This approach is designed to establish clear temporality in the relationship between health and housing by isolating true “shocks,” which can then be leveraged in an event study design. Enrollees were included if they were between the ages of 3 and 62 and continuously enrolled (10+ months enrolled over each 12-month period) for the 4 years around the shock. They needed to report at least one address within NYC to be included in the analysis, as details about addresses (shelter lists, housing quality data, etc.) are only available in NYC. To isolate true health shocks that are plausibly random in timing, two exclusion criteria were enforced: (1) enrollees were excluded if they had a birth-related hospitalization, and (2) enrollees were excluded if they had more than a 50 % increase in mean health care utilization (sum of emergency and outpatient visits) in the two quarters preceding the hospitalization relative to the prior 6 quarters. These conditions eliminate hospitalizations that were anticipated.

A matched comparison group of enrollees with no hospitalization for at least two years, continuous enrollment for four years, and at least one address in NYC, was identified, with a randomly-chosen date as their “exposure.”^3^ This produced a large pool of potential comparators (*n* = 1309,884; or, virtually all long-term Medicaid enrollees in NYC without a hospitalization). Exact matching was used to select matches based on age category, borough of residence as of one year prior to the “exposure” date, enrollee-reported race/ethnicity, enrollee-reported sex, year of the shock (or “pseudo-shock” for comparators), historic enrollment in Medicaid (4 years prior), and Medicaid eligibility reason as of one year prior (e.g., foster care, SSI-eligible, single childless adult, etc.).^4^

Reasons for inpatient admission were assigned clinical classification software (CCS) categories, which are groupings designed by the Agency for Healthcare Research and Quality that group diagnoses into analytic units like “asthma,” “mood disorders,” or “myocardial infarction.” These categories were further collapsed for heterogeneity analyses into broader categories to encompass (1) injuries, (2) substance use disorder-related conditions, (3) mental health-related conditions, (4) cardiac or stroke-related admissions, (5) other urgent admissions (e.g., diabetes complications, appendicitis, seizures, etc.), and (6) non-urgent admissions (bariatric surgery, joint replacements, etc.). A mapping of CCS categories to the broader groupings is shown in Appendix Table A1.

#### Outcomes

**Appendix Fig. A1.**
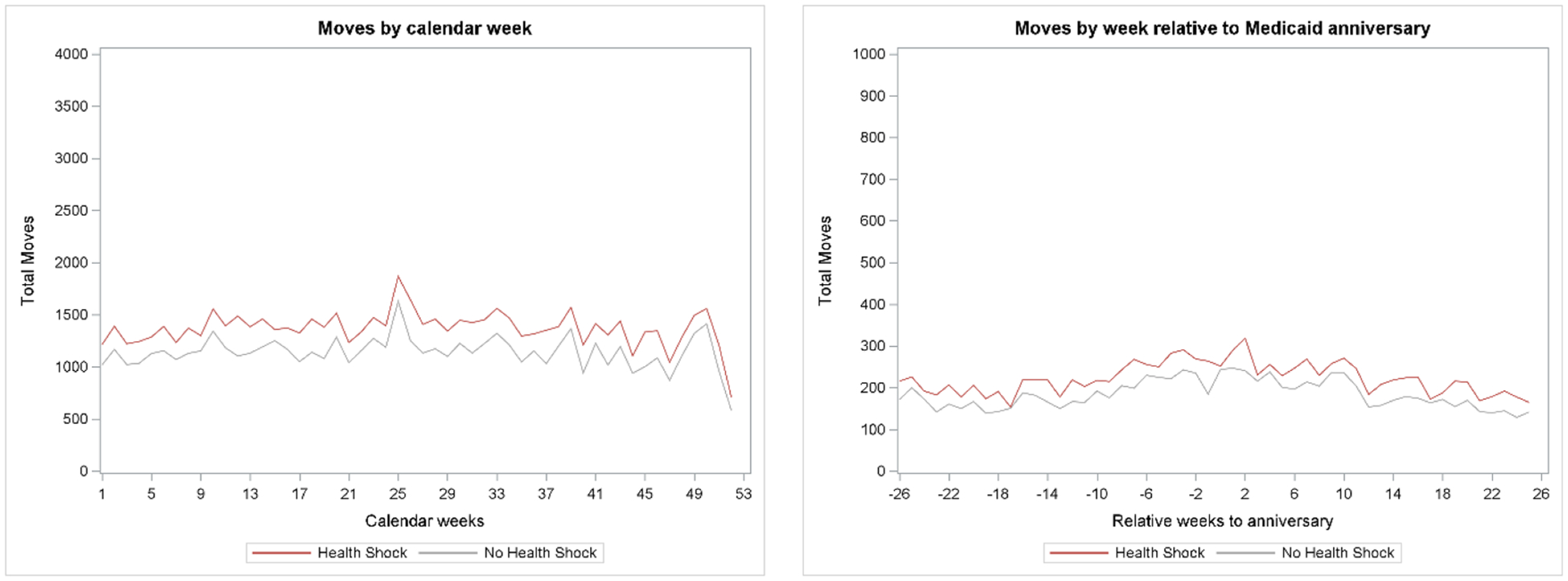
Move counts by calendar week and week relative to enrollment anniversary. *Notes:* These graphs plot the raw total number of moves by calendar week and by weeks relative to enrollment anniversary for enrollees who did and did not experience health shocks. There are 237,199 enrollees in each group.

The primary outcome for this study is the number of reported address changes over 4 years around the shock, counting from the day of hospitalization. Addresses in the data set are regularly updated and verified through several mechanisms, including every time enrollees or caseworkers engage with Medicaid, their managed care plans, or other State-administered social services, and at regular re-enrollment verification windows (annually/semi-annually depending on Medicaid eligibility category). As New York State relied on physical mail in this study period (pre-2020); requires regular contact with enrollees to relay and verify coverage information; and has implemented thorough procedures and requirements for maintaining accurate contact information through managed care plans (The Coverage Learning Collaborative, 2021), these fields have high fidelity. Research has found high concordance between Medicaid addresses and self-identified housing status, like homelessness (Pourat et al., 2023; Vickery et al., 2018). Histograms showing reported move timing by calendar date and by Medicaid enrollment anniversary are available in Appendix Fig. A1. Both figures demonstrate consistent rates of address updates over time relative to enrollment anniversaries and throughout the calendar year (i.e., no bunching), with a very modest rise in address updates in the weeks directly around annual enrollment anniversaries. This supports the reliability of using the administrative addresses to measure mobility, as addresses appear to be updated consistently and constantly. If addresses were only collected through annual campaigns (e.g., every January), or only collected at the time of re-enrollment, we would expect larger peaks and troughs in these figures.

Addresses were cleaned and collapsed to account for typographical errors and errant administrative updates to avoid overcounting moves (Fecht et al., 2020). They were geocoded to the building level using an NYC-specific geocoder (NYCgbat) to obtain a unique “borough-block-lot” (BBL) identifier to which building-level details are tied (NYC Department of City Planning, 2021). For the primary models, moves are aggregated into 3-month windows counting from the day of the health shock onward (referred to as “quarters”).^5^ An address change was counted any time the enrollee’s building identifier changed. This captures the most encompassing definition of residential mobility, free from assumptions about the nature of the move. It also represents an advantage over prior work which has only been able to capture specific types of moves, like moves into shelters or formal evictions in court.

However, several secondary outcomes were also measured to account for the fact that not all moves are “negative moves.” While a large share of moves among low-income populations are “negative” or forced (Desmond and Shollenberger, 2015; Phinney, 2013), people experiencing illness might also experience “positive” moves, such as moving in with family temporarily or to accommodations more suitable to their condition (e.g., stair-free). As one way to attempt to shed light on “negative” moves, I created a binary indicator flagging those with 2+ moves per quarter (between distinct addresses) as an indicator of extreme instability. Examining extreme mobility using high-frequency administrative data is policy-relevant, as these groups are highly vulnerable to long-term instability; are likely facing the largest barriers to securing stable housing; and have not been well-described in prior work using survey data with multi-year gaps between waves. While prior work has often defined “instability” as at least 3 moves in one year, this has largely been driven by the frequency of waves in survey-based data, with many researchers and agencies indicating interest in even more frequent mobility as an indicator of housing insecurity (Cutts et al., 2011; Ha et al., 2020; Kang, 2021; Office of Disease Prevention and Health Promotion, (n.d.)). Measuring 2 or more moves per quarter sheds light on this phenomenon by converting the construct of residential instability in prior survey-based studies into a quarterly metric amenable to shorter-run event study analyses. Empirically, this outcome overlaps substantially but not completely with a homelessness indicator described below, with 51 % of all enrollees flagged as “extremely mobile” also being flagged as homeless (compared with 4 % flagged as homeless in the non-mobile group). This suggests it is capturing a highly relevant but still distinct housing instability concept.

As another indicator of negative moves, I calculated the share of people reporting an address linked to explicitly “unstable” situations typically indicative of homelessness (shelter addresses; street homelessness based on validated indicators like “undomiciled” or General Post Office locations used by individuals without an address; temporary “cluster” sites; headquarters of known social organizations or places of worship; or jail) (Pourat et al., 2023; Vickery et al., 2018; Zech et al., 2015). NYC caseworkers are instructed to use the General Post Office addresses and “undomiciled” term when assisting applicants with Medicaid or other social service forms (New York City Family Independence Administration, 2003), and places of worship or other social service organizations report listing their own address when assisting people experiencing homelessness enroll in benefits (Pourat et al., 2023; Vickery et al., 2018).

Finally, I created indicators to characterize the quality of the moves. The number of housing violations per 100 units was created by summing all city-issued violations at each BBL, including absence of heating and water, broken windows and elevators, mold and pests, and illegal construction or noise, among other issues.^6^ I also measured Census tract-level poverty rates for the at each quarter. I excluded the poverty rates of addresses of shelters or social service organizations, which are often located in higher income areas. Finally, I created measure of mean distance moved, calculated as the as-the-crow-flies distance between the addresses at the end of each quarter.

### 3.2. Statistical analyses

As the main empirical specification, I measured the change in housing outcomes in the high-frequency panel data using non-parametric event studies, which capture changes after the health shock, relative to expected rates based on a comparator group over the same period. As described above, quarters are calculated as 3-month periods relative to the date of the health shock (i.e., they do not correspond to calendar-year quarters). The quarters are centered on the exact date of the shock as time 0. Comparison group enrollees had to have had no hospitalization for at least two years, continuous enrollment for four years, and at least one address in NYC. They were assigned a randomly-selected date in their eligibility period as their “pseudo-shock” event date, and they were exact-matched to enrollees who did experience a health shock across a number of demographic and enrollment characteristics (see Section 3.1 Data and Sample for more detail).

The event study specification uses dummy indicators for each quarter relative to the pre-shock quarter to flexibly model quarter-specific effects. The specification is as follows:

(1) $$y_{\text{it}}=\mu_{i}+\sum_{k\neq -1}\theta_{k}\left(1\left\{{\text{RelQuarter}}_{\text{it}}=k\right\}{\text{Group}}_{i}\right)+\gamma_{t}+\epsilon_{\text{it}}$$

This returns quarter-specific coefficients for the change in housing outcomes relative to the difference between the health shock group and their comparators in quarter t-1 (the reference quarter). The individual fixed effects capture time invariant confounders like race or sex, and calendar-year fixed effects absorb NYC-wide time-varying confounders, like general fluctuations in rent prices or changes to the address data quality. Sensitivity analyses described below integrate additional time-*varying* confounders, like the quarterly count of health system visits made by the enrollee. Standard errors in all models are clustered at the enrollee level.

### 3.3. Tests of assumptions and sensitivity analyses

The core assumption of this approach is that, in the absence of a health shock, the trajectory of housing outcomes would have remained steady, as modeled by the comparators. This strategy requires that the health shock group and comparator group be on a similar trajectory (parallel trends) prior to the event. To build evidence supporting this assumption, we can examine the pre-shock event study coefficients: if they are close to 0, this indicates that the health shock and comparator groups were trending similarly up until the shock. It then follows that we could have expected those trends to have continued in the absence of the shock, supporting our use of the comparator group to build the counterfactual.

This approach also requires that no other confounding shock occurred simultaneously that would cause both housing disruptions and health crises. It is possible that events that are not directly observable in these data – such as a divorce or job loss – cause both a health crisis and a move. I test for the potential influence of divorces or other family relationship changes by assessing whether the number of people on a Medicaid case changes just before the health shock, indicating a change in family composition. Job loss is not directly testable in these data, although qualitative and sociological research suggest that job loss most typically *follows* a health shock rather than *precedes* it (Doran et al., 2019; Kushel et al., 2023; Shinn and Khadduri, 2020). Still, I examine trends in Medicaid enrollment as described below, which may also be a proxy for capturing labor market changes.

This research also relies on accurate measurement of mobility. The central advantage of this research is the novel use of high-frequency administrative address data to capture residential instability in real time, rather than the periodic surveys with 1–3 year measurement gaps used in prior literature. This high-frequency data strengthens the case that the timing of the health event precedes residential instability. However, I also conducted two tests that further support the use of the administrative addresses as measures of genuine residential moves and help rule out administrative artifacts as an explanation. If people are simply engaging with providers and Medicaid more during and after a health event – and those providers are regularly asking for address verification – an increase in “moves” might appear, even though reported changes are merely due to differences in measurement frequency. First, I use an alternative measure of housing instability that does not rely on administrative addresses at all. I leverage newly available diagnostic codes that capture housing insecurity, known as “Z-codes.”^7^ While these housing-related Z-codes vastly under-coded and can only be observed in claims data if individuals are actively utilizing care (Pourat et al. 2023), seeing a similar break in trend as the address-based outcome would lend credibility to the main results. These diagnostic codes are recorded on a claim if a patient tells their provider they are experiencing housing-related problems, acting as a measure with high specificity and positive predictive value (i.e., few false positives). This analysis controls for the total number of health care visits a person had per quarter, to account for the fact that a change in health induces more visits (and thus documented diagnoses) in general.

As a second check of the reliability of using administrative addresses to capture moves, I separately examined individuals admitted for sudden but non-urgent admissions like bariatric surgery or joint replacements. The role of the health event would not be expected to change housing stability (but *would* be expected to increase their address update frequency if the mobility measure was purely mechanical). Finding no effect of these admissions on housing instability would provide compelling evidence that the address-based move metrics are reliably capturing genuine moves, rather than simple administrative updates when enrollees engage with Medicaid and their providers during spells of illness.

I conducted several other sensitivity analyses to assess the stability of the results. First, I tested the model when limited to those that arrived via emergency transport only, to isolate true shocks from less urgent stays. I also re-estimated effects excluding moves to and from health care facilities (e.g., nursing homes, psychiatric facilities), as these moves may simply reflect something about the health event directly rather than capturing *consequences* of a health event to one’s housing stability. Finally, I re-estimated the main model controlling for the total number of health care visits per quarter, as an additional check against the threat that people’s addresses may be more likely to be administratively updated if they are interacting with Medicaid or providers more frequently. However, it should be noted that controlling for this variable may also effectively “control away” some of the genuine effect of worse health on residential mobility in the post-shock period: if “total visits” also captures genuinely worse health, and we believe worse health corresponds to a higher likelihood of experiencing health-induced housing instability, we may be estimating an overly-conservative treatment effect when incorporating this control variable.

I also tested whether the requirement of continuous enrollment introduced bias or external validity issues by examining whether health shocks induced differential Medicaid disenrollment. If people who remain on Medicaid after a health shock are also more likely to experience subsequent housing instability, then relying on continuous enrollment may overestimate the impact of health shocks. Conversely, if people remaining on Medicaid are less likely to experience subsequent housing instability, this approach may underestimate the impact. I cannot observe housing stability rates for those who disenroll. However, assessing whether the shock alters mean months enrolled 2 years later – relative to comparators – is one test of the magnitude of this issue. This analysis also sheds light on whether there are contemporaneous labor market changes for the population experiencing health shocks that could confound the results, as we might hypothesize that people with disrupted employment might be disproportionately likely to remain on Medicaid long-term.

### 3.4. Heterogeneity and mechanisms

To examine effect heterogeneity and test theories about mechanisms, I used the same specification as Eq. (1). However, here, the “group” indicators flag different subgroups of the given characteristic (e.g., diagnosis category), to capture the difference in effect size across levels of the characteristic of interest. In the descriptive heterogeneity results, only raw estimates of the differences in effect sizes across groups are calculated without any additional control variables. The characteristics I examine in these unidimensional heterogeneity tests include reason for admission (by diagnosis category), age, race/ethnicity, type of pre-shock housing (subsidized vs market rate), and length of stay in the hospital.

In analyses examining potential policy *mechanisms* – rather than just describing heterogeneity – I control for confounders relevant to each proposed mechanism. Confounders in this setting could be any characteristics associated with the mechanism of interest and are specified in the results section for each analysis. For example, people who secure subsidized housing may be more likely to be older, and older individuals may be more likely to experience housing instability after a health event. I adjust for these confounders by including additional terms interacting of the quarter dummies and confounder.

## 4. Results

Overall, 237,199 enrollees experienced a health shock. Table 1 shows that the group was demographically diverse, with 8 % Asian, 28 % Black, 30 % Hispanic, and 14 % White enrollees, and 19 % were children. Because the comparators (*n* = 237,199) were exact-matched on demographic characteristics, they did not meaningfully differ on any characteristics (Table 1).

Reasons for admission during the health shocks varied widely, with no single CCS category accounting for >5 % of admissions overall across the whole sample. Among children, reasons for admission were more concentrated, with asthma admissions alone accounting for 14 % of shocks. Among adults, however, the top five causes together accounted for only 19 % of admissions. Approximately 16 % of all adult admissions were mental health- or SUD-related. Bariatric surgery – the majority of cases with a diagnosis of “morbid obesity” and weight-related diagnoses – was common, comprising 3 % of adult admissions. Because procedures like this may not be “shocks” in the same way that strokes, mental health crises, or injuries may be, I excluded them in sensitivity analyses.

### 4.1. Event study results

**Fig. 1.**
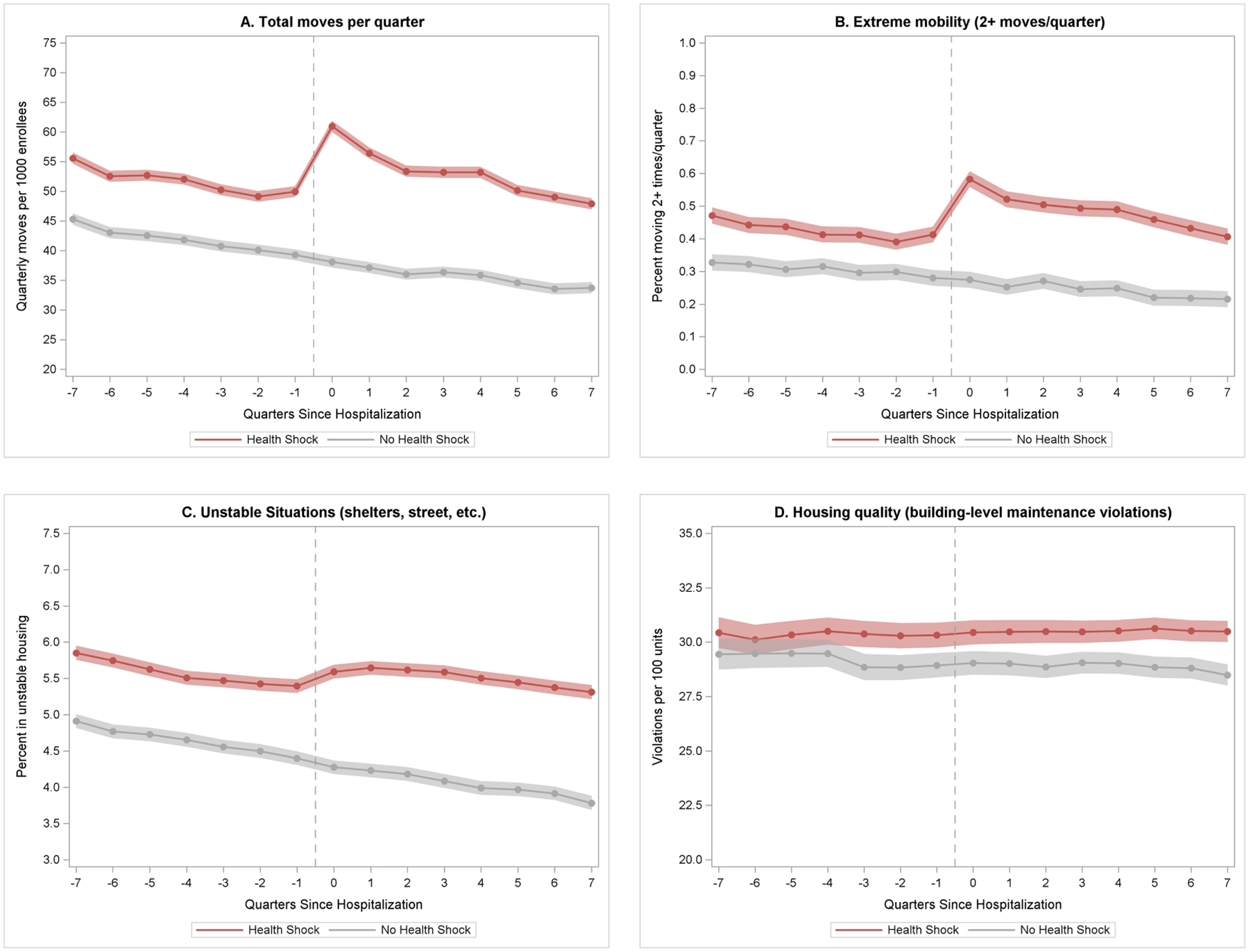
Quarterly outcomes before and after health shock. *Notes:* The colored points in Fig. 1 show the rates for each outcome in the quarters before and after the health shock (or pseudo-shock) for the two groups. The points are the means of each outcome, obtained by regressing each outcome on an indicator for each quarter and an indicator for each calendar year. Standard errors for the points are clustered at the enrollee level. Maintenance violations data was only available 2014–2019. All other data spans the entire period from 2010–2019. Values are scaled per 100 or per 1000 for interpretability. There are 237,199 enrollees in each group.

Fig. 1 shows the raw trends for all 4 main outcomes for both the health shock and comparator groups. The jump at the time of the health shock is visible for the total moves, extreme mobility, and instability outcomes. Fig. 1 also emphasizes a stable, downward trend in mobility and instability for the comparison group. This downward trend is likely due to the fact that enrollees often enter Medicaid at a time of new or acute poverty and gradually see their lives stabilize. Fig. 1 highlights how this trend toward improvement is substantially interrupted when a health crisis occurs.

**Fig. 2.**
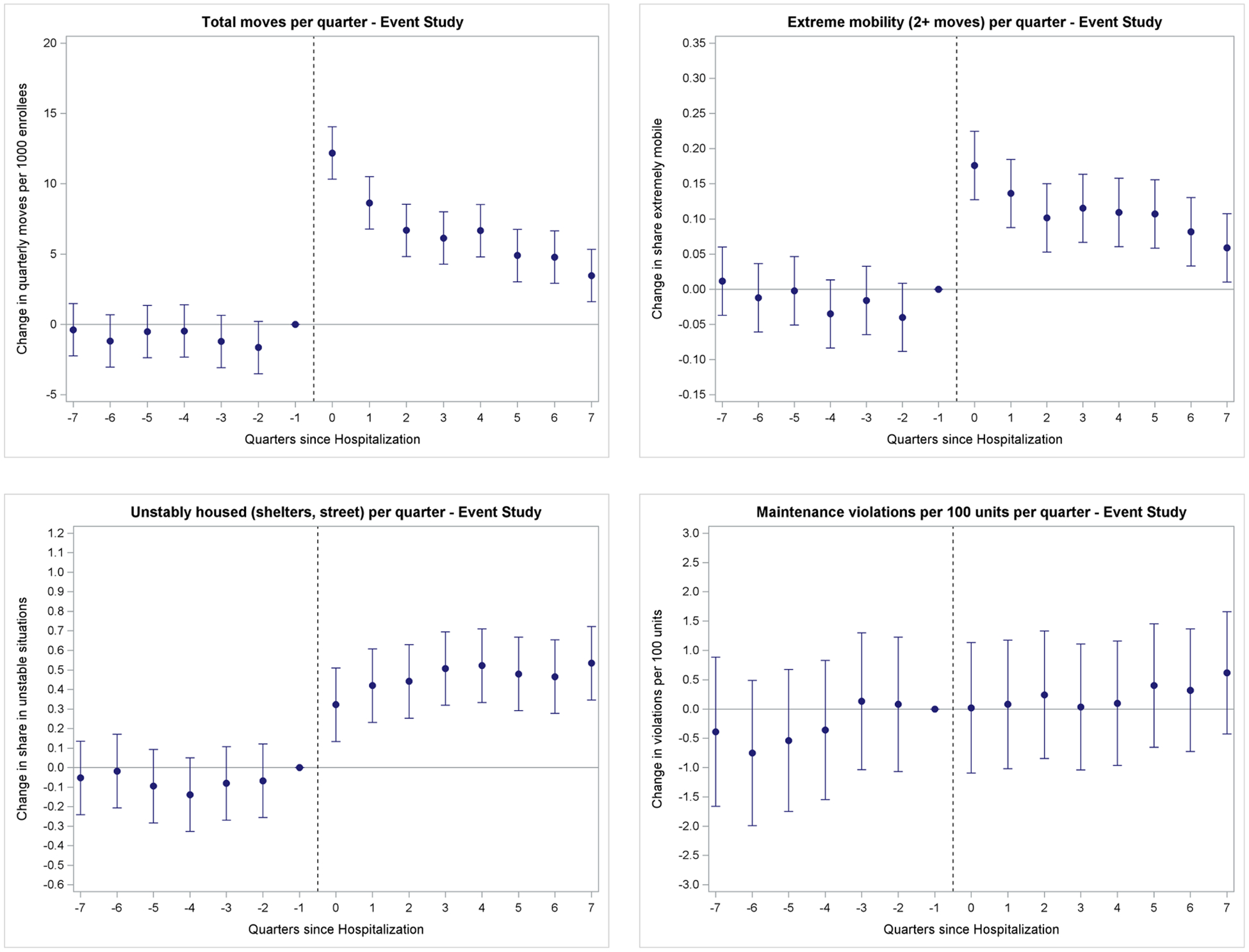
Event study estimates of quarterly outcomes. *Notes:* Fig. 2 shows the event study estimates estimated from Eq. (1). Standard errors for the points are clustered at the enrollee level. Maintenance violations data was only available 2014–2019. All other data spans the entire period from 2010–2019. Values are scaled per 100 or per 1000 for interpretability. There are 237,199 in both the health shock and comparator groups.

The estimate from the event study estimation of the change in total quarterly moves by the first quarter after the shock is 12.2 (95 % CI: 10.3, 14.0) – a relative increase of 25 % from the expected rate. As shown in the effect estimates in Table 2 and Fig. 2, the quarterly move rate immediately jumps to a higher level and remains above the expected level for over two years, still 14 % above expected by the 5th quarter after the shock. Nationally, Medicaid enrollees experience approximately 6.5 million non-birth hospitalizations annually (Liang et al., 2020), implying that adverse health events may induce 80,000 additional residential moves in the Medicaid population in the first quarter after their hospitalization alone (or about 240,000 additional moves total over the first post-shock year). Likewise, the share who are extremely mobile (making 2+ moves/quarter) increases by 0.2 percentage points (pp) (95 % CI: 0.1, 0.2) by the first quarter post-shock, or a 45 % increase in this rare but policy-relevant outcome. It similarly remains elevated above expected levels for the following two years.

There is also evidence of an increase in the share of people in particularly acute situations that would traditionally be considered homelessness (e.g., in shelters or on the street, or reporting a social service organization or jail as the address), although the magnitude is only a 0.32 (95 % CI: 0.13, 0.51) pp increase (6 % relative increase) in the first post-shock quarter. However, this grows slightly over the following quarters, reaching a 10 % relative increase from the expected level by the 5th quarter after the shock. Using a stricter definition of homelessness (street homelessness and shelter addresses only), the effect size is similar: a 0.25 percentage point increase in the first quarter (95 % CI: 0.10, 0.40), which is an 8 % relative increase from the expected rate of this outcome. Scaling this up to the national Medicaid population, this implies an increase of around 20,000–30,000 cases of homelessness triggered by health shocks in the first post-shock quarter.

There is no evidence of a change in the quality of housing as measured by building violation rates or tract-level poverty rates. Even though the numbers of moves increase, the moves are not disproportionately toward worse housing (0.0 change in violations/100 units, 95 % CI: −1.1, 1.1) or to poorer neighborhoods (−0.1 change in poverty rate of neighborhood, 95 % CI: −0.1, 0.1). One likely explanation is that the destinations of people experiencing housing instability are often short stints with better-off friends or relatives – e.g., couch surfing or other temporary agreements – or unconventional locations like motels and hostels (Office of Community Planning and Development, 2023). Additionally, the quality of the large majority of low-income housing in NYC is uniformly poor, and therefore the range of housing quality is restricted when many enrollees are already living among the lowest-quality housing available. For these reasons, a null effect of a health shock on the quality of housing people move to may not be particularly surprising. There is some evidence that, conditional on moving, enrollees experiencing a shock move slightly farther than would be expected in the absence of a shock, but this difference does not appear until several quarters after the shock (by the 5th quarter: 0.4 additional miles, 95 % CI: 0.1, 0.7, 9 % relative increase).

### 4.2. Sensitivity analyses results

Results from all of the sensitivity analyses are summarized in Table 3. As expected, the impact of health shocks was larger when removing non-urgent procedures (like bariatric surgery and joint replacements), for a 35 % relative increase (19.0 additional moves per 1000 enrollees in the first quarter, 95 % CI: 16.3, 21.6). The larger estimate that is obtained when excluding non-urgent admissions may give a more realistic sense of the true impact of health “shocks” on housing stability. Limiting to arrivals via ambulance results in a similar effect size to the main analysis (10.7 additional moves per 1000 enrollees; 95 % CI: 7.4, 13.9).

Using an outcome that excluded moves to and from health facilities like nursing homes resulted in a similar relative increase as the main analysis (21 % increase), indicating that rehabilitation-specific moves do not explain the observed relationship. Adding total quarterly health care visits as a covariate results in a slightly attenuated effect size. While this model accounts for the fact that a health shock may simply present additional opportunities for enrollee address updates, it may also be an overly conservative specification: total visits are also likely correlated with genuine severity of the health shock and may therefore “control away” some of the true relationship between health shocks and residential instability in the post-shock period.

**Appendix Fig. A2.**
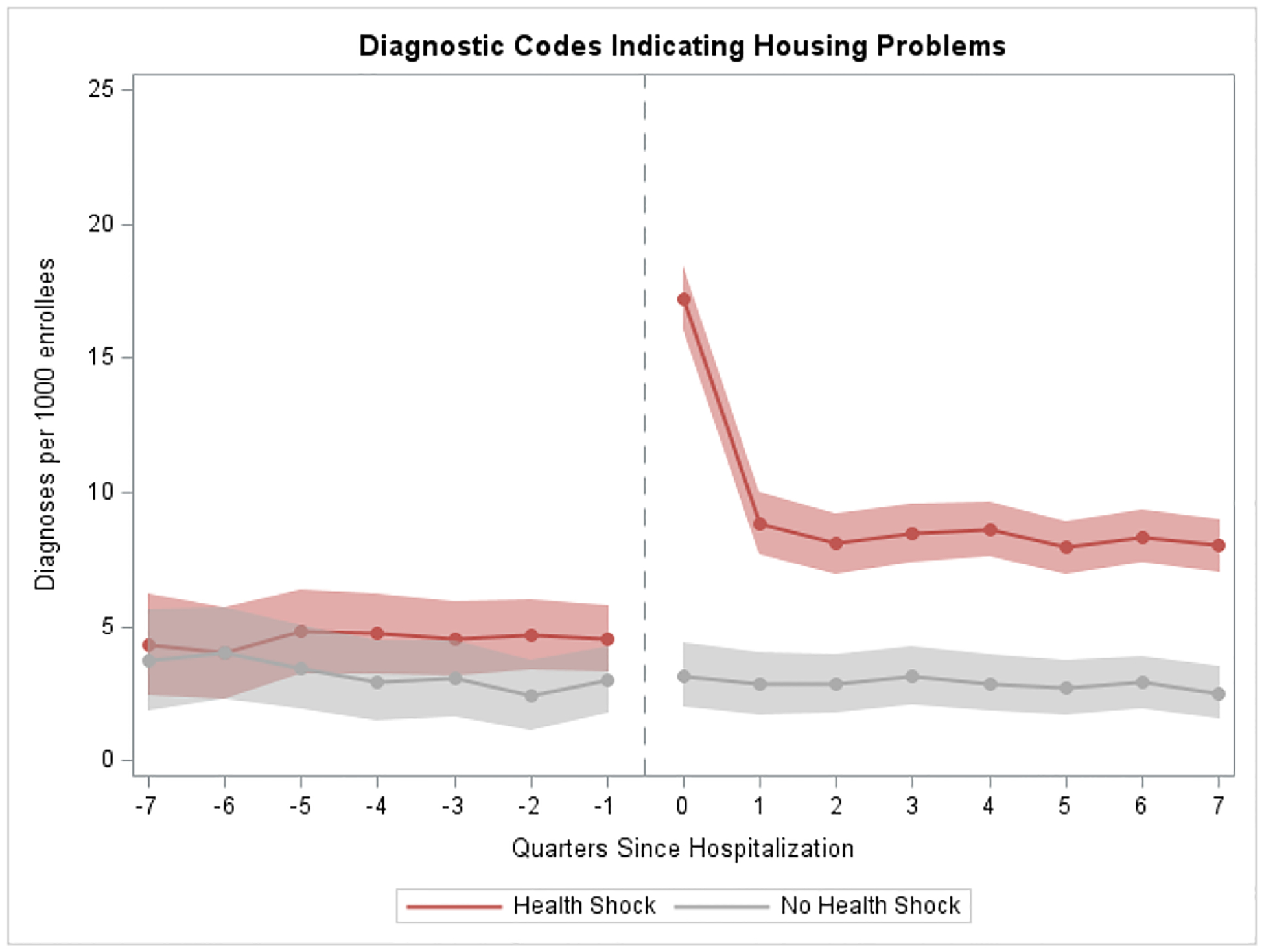
Quarterly housing instability diagnosis code rates before and after health shock. *Notes:* The colored points show the rates for housing instability ICD-10 diagnostic codes in the quarters before and after the health shock (or pseudo-shock) for the two groups. The points are the means of the outcome, obtained by regressing each outcome on an indicator for each quarter and an indicator for each calendar year, as well as controlling for the quarterly count of total health care visits. Standard errors for the points are clustered at the enrollee level. Diagnostic code data was only available 2015–2019. Values are scaled per 1000 for interpretability. There are 240,982 enrollees in each group.

When using a definition of housing instability based on housing instability diagnostic codes rather than addresses, I found a very large increased risk in the first post-shock quarter. This risk then decreases in subsequent quarters, but remains about twice as high as the expected rate (in the first post-shock quarter, there are 12.5 additional instability diagnoses per 1000 people, 95 % CI: 10.1, 15.0, 269 % relative increase; by the second post-shock quarter, the relative difference is 109 % higher than expected). The model adjusts for the quarterly number of total health system visits (and thus opportunities for new diagnoses to be recorded). The similar pattern in housing instability between this analysis and the primary outcome supports the main results and suggests persistence of some degree of instability over the long term. The graph of this result is available in Supplemental Appendix Fig. A2.

**Appendix Fig. A3.**
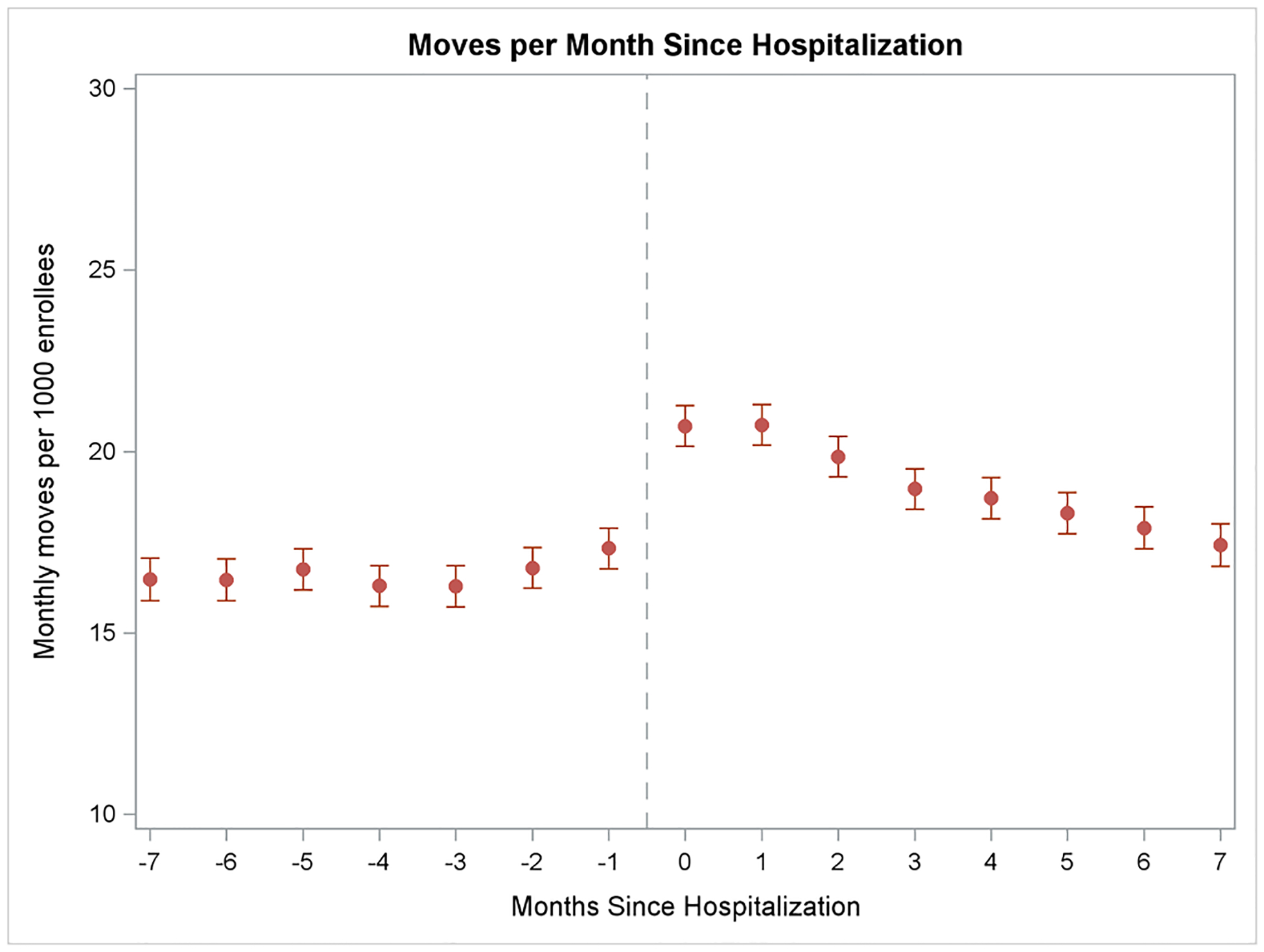
Moves by quarter since hospitalization aggregated at the monthly level. *Notes:* The colored points show the rates of moves in the months before and after the health shock. The points are the means of each outcome, obtained by regressing each outcome on an indicator for each quarter and an indicator for each calendar year. Standard errors for the points are clustered at the enrollee level. Values are scaled per 1000 for interpretability. There are 237,199 enrollees used to produce this figure.

Aggregating the outcome into months since the shock (rather than 3-month “quarters”) shows a similar jump in the months following hospitalization of 19 % (Appendix Fig. A3), indicating that using a quarterly aggregation in the primary specification is not obscuring complex patterns or pre-trends. The monthly aggregation also demonstrates that moves are equally spread across the first three post-shock months that comprise the first post-shock quarter, rather than clustering just in the month immediately following the hospitalization. This consistent spacing supports the assumption that these are genuine moves: if, in contrast, all of the moves had clustered immediately in the first month after the hospitalization, one may worry that the address changes were simply administrative updates occurring *because* enrollees are using the health system during a health shock.

To understand the implications of requiring continuous Medicaid enrollment for inclusion in the analysis, I also examined whether post-shock Medicaid enrollment patterns differed for people who experienced a health shock and those who do not. This analysis included any enrollees with 2 years of continuous enrollment *only prior* to the “shock.” In a model adjusting for race, sex, and age, the total number of months enrolled 2 years after the shock was slightly lower for those who experienced a health shock versus a comparator group who did not: 10.2 versus 10.5 months enrolled (out of 12), or a −3.5 % relative difference. While statistically significant, this difference is unlikely to be of a large enough magnitude to substantially bias the findings. Additionally, disenrollment would need to be systematically correlated with post-shock mobility for this to bias the main findings.^8^ The fact that there are no large or systematic differences in enrollment for those with and without a health shock also supports the notion that job loss or labor market changes are not likely confounding the observed relationship. If a Medicaid enrollee lost a job prior to falling ill, we would likely expect post-shock enrollment to be higher than for those without a shock (as they would need to rely even more heavily on safety net programs like Medicaid in the wake of a job loss). The negligible difference in enrollment suggests that job loss as a confounder may not be a significant concern.

**Appendix Fig. A4.**
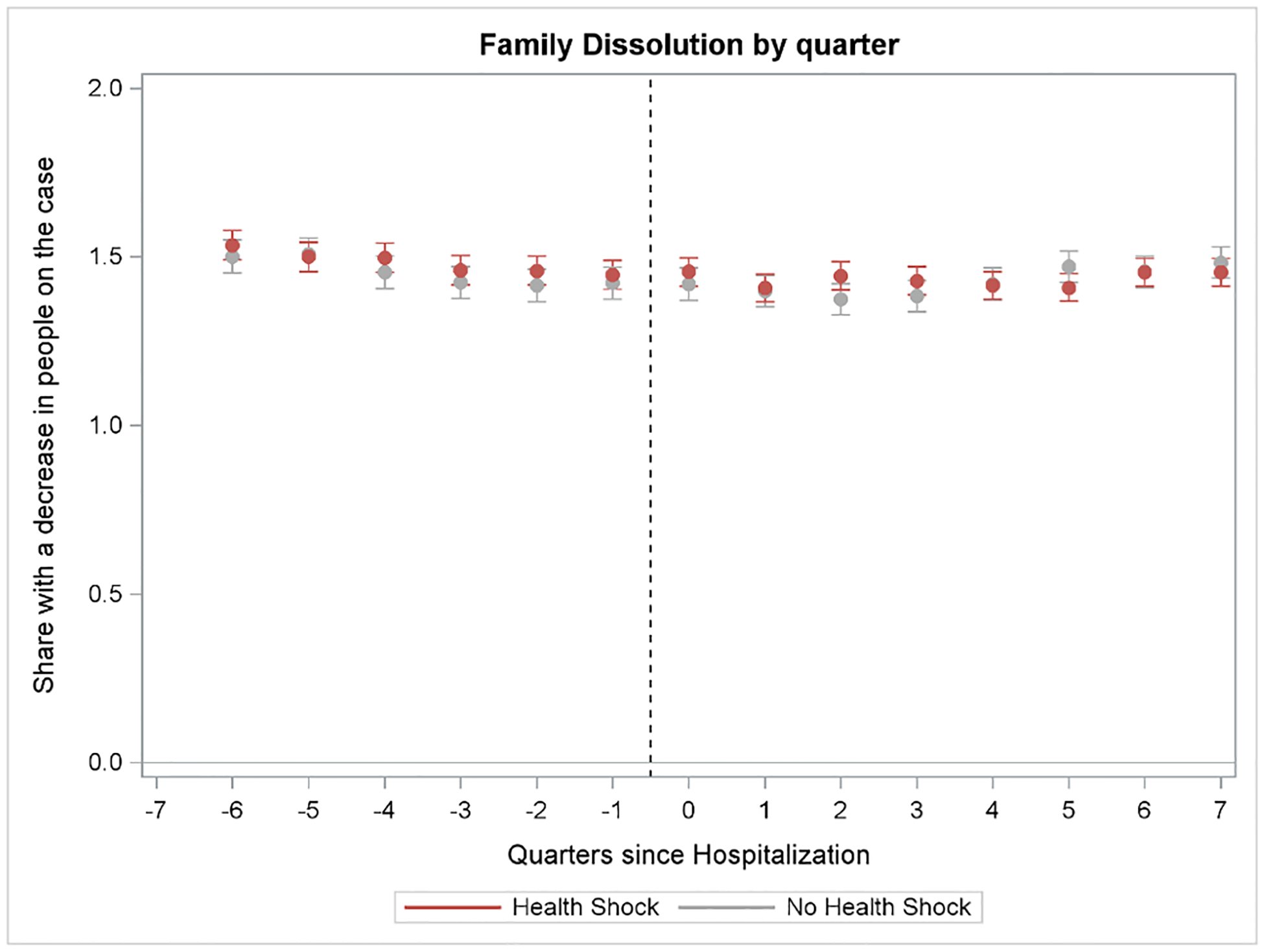
Rates of family dissolution (decrease in number of members on medicaid case) by quarter. *Notes:* The colored points show the rates of family dissolution in the months before and after the health shock. Family dissolution is defined as a decrease in the number of people on the Medicaid case. The points are the means of the outcome, obtained by regressing each outcome on an indicator for each quarter and an indicator for each calendar year. Standard errors for the points are clustered at the enrollee level. Values are scaled per 100 (i.e., percent) for interpretability. There are 237,199 enrollees in each group.

In these data, I can also observe changes to the number of people on a Medicaid case, which may be a proxy for divorce or family dissolution. Family dissolution could theoretically confound the relationship, as it may cause a health crisis (e.g., mental health crisis, stress-induced cardiovascular event) and also cause housing instability. Using an indicator for whether the number of people on a Medicaid case decreased by quarter, I do not find evidence that divorces preceded or coincided with the health shocks and housing instability: the percentage point change in the quarterly share of households with this indicator remains at 1.4 to 1.5 percent for both the treated and comparator groups for all quarters around the health shock (*p* > 0.2 for all quarters, relative to the quarter before the shock; Appendix Fig. A4).

### 4.3. Heterogeneity and mechanism analyses

**Fig. 3.**
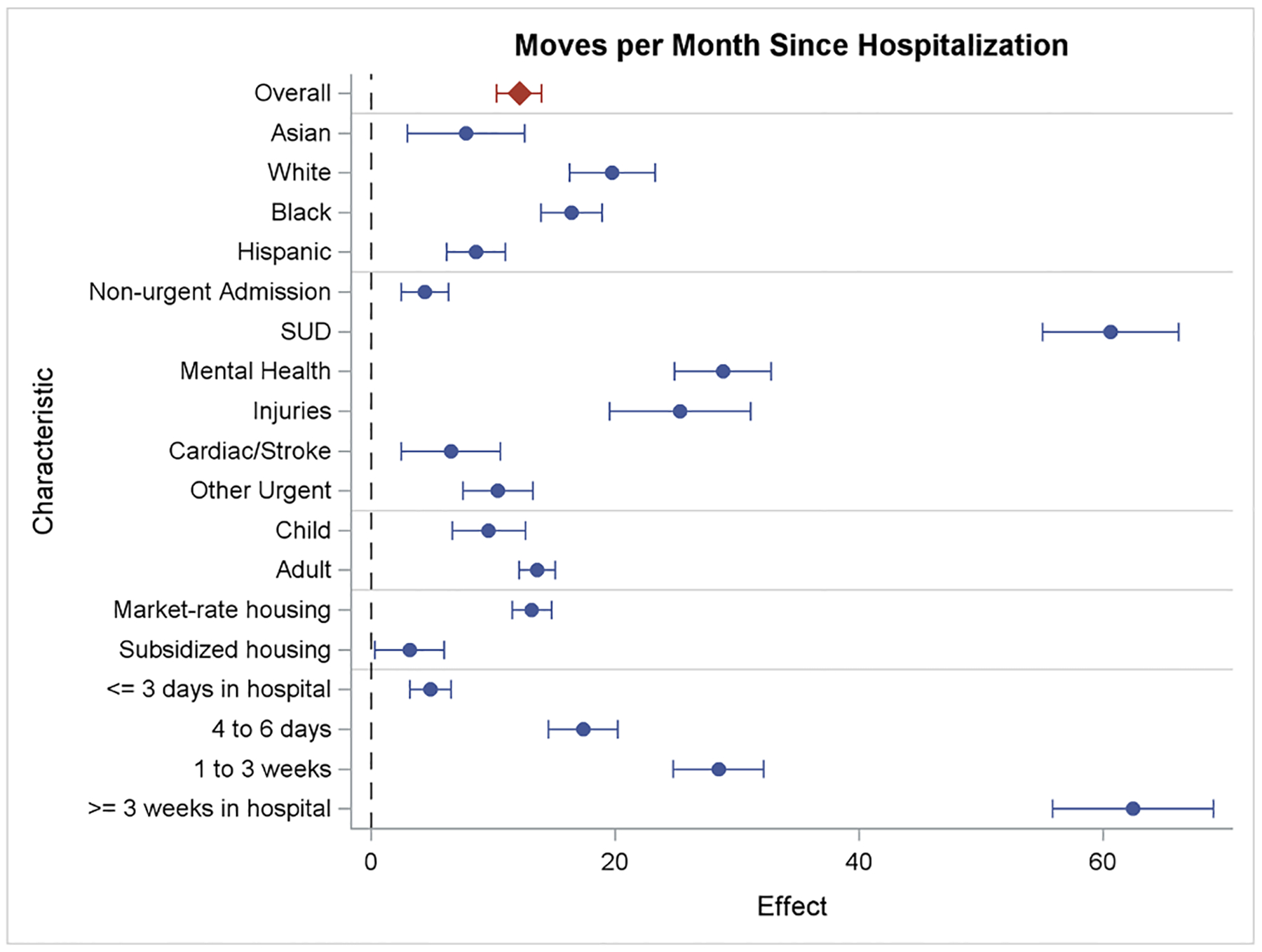
Unidimensional heterogeneity in effect size (“Total Moves” Outcome). *Notes:* Fig. 3 shows unidimensional (unadjusted) heterogeneity estimates for each of the listed characteristics and the change in the trend at the time of the health shock. These estimates are limited to the enrollees who did experience a health shock. They are estimated via the event study equation (Eq. (1)), interacting the indicator for the given group characteristic with the quarter indicator. The effect is measured as additional moves per 1000 people induced by the health shock. The overall effect, indicated by the red diamond, is 12.2 additional moves per 1000 people after a health shock. SUD means Substance Use Disorders, including alcohol use disorder. See appendix Table A1 for mapping of diagnostic categories to the broader analytic groups shown here. There are 237,199 enrollees total in these analyses.

The primary results demonstrate that health events increase risk of housing mobility and homelessness for Medicaid enrollees overall, yet this relationship likely varies in magnitude by household and policy factors. Fig. 3 shows unidimensional heterogeneity in the increase in total moves across several characteristics. Those in subsidized housing saw virtually no relationship between health shocks and housing instability and enrollees of Asian or Hispanic race/ethnicity saw substantially smaller effects. Conversely, long lengths of stay (LOS) and stays for injuries, SUD, and mental health saw larger effects.

Fig. 3 also highlights the fact that there is a substantially smaller increase in moves for people with non-urgent admissions (like bariatric surgery or joint replacements) – specifically an increase of only 4 moves per 1000 enrollees, versus an increase of 8 to 60 moves for other conditions (injuries, cardiac/stroke, other urgent admissions, mental health, and substance use disorder). This supports the notion that the address updates are not just artifacts of an increase in engagement with administrative systems as enrollees engage with Medicaid and providers during their illness. Further, the fact that shocks for children generate a similar change in housing stability to adults supports the idea that these health events are not primarily driven by confounding factors like divorce or job loss: health shocks for children are less likely to be the result of divorce or job loss, but we still see a large impact on the household’s mobility.

**Fig. 4.**
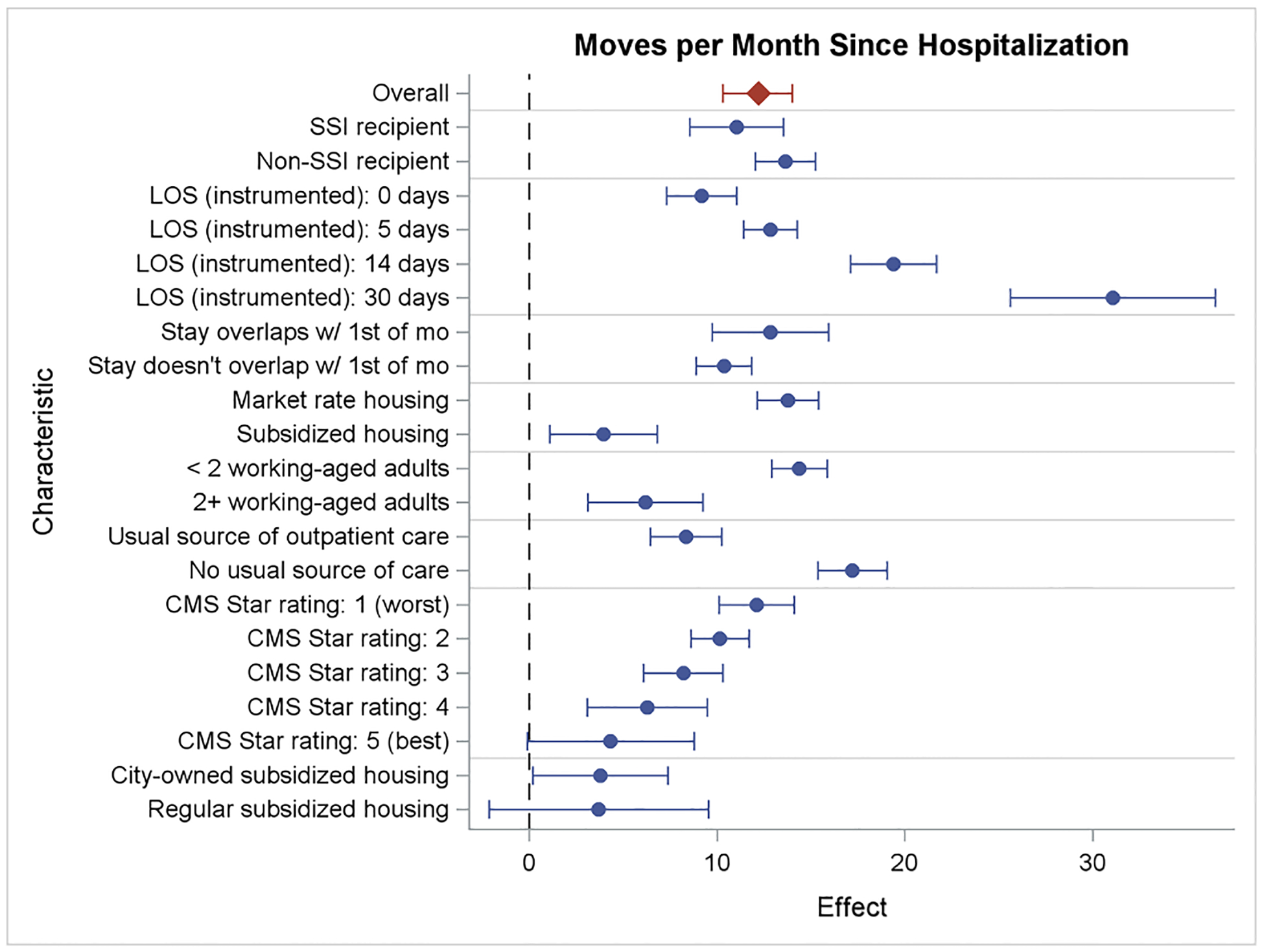
Heterogeneity analysis of policy-relevant mechanisms (“Total Moves” Outcome). *Notes:* Fig. 4 shows heterogeneity estimates for each of the listed characteristics from models controlling for potential confounders associated with both the policy-relevant characteristic and the risk of housing instability. These estimates are limited to the enrollees that did experience a shock. The estimates are produced by Eq. (1), interacting the indicator for the given group or policy characteristic with the quarter indicator. In the case of length of stay (LOS), jackknife instrumental variable estimation is used to create a plausibly exogenous proxy for Length of Stay (the random variation in how long facilities tend to keep patients for a given condition). For the analysis of heterogeneity by whether the stay overlaps with the first of the month, only stays shorter than 30 days could be used. The effect is measured as additional moves per 1000 people induced by the health shock. The overall effect, indicated by the red diamond, is 12.2 additional moves per 1000 people after a health shock. SSI means supplemental security income. LOS means length of stay. CMS means the Centers for Medicare and Medicaid Services. There are 237,199 enrollees total used in the models producing these estimates.

However, this unidimensional variation alone does not isolate potentially influential policy mechanisms. I therefore also tested heterogeneity across three categories of policy-relevant factors, by identifying measurable policy-relevant mechanisms and either controlling for confounders or by leveraging instrumented versions of variables: (1) income or rent disruptions, (2) implicit insurance conferring resilience, and (3) adequacy of the safety net. The following sections investigate each of these buckets in detail, and the findings are summarized in Fig. 4. It is important to note that the policy mechanism analyses in this paper should be viewed as exploratory, as they may still be confounded by factors associated with both the given characteristic and the likelihood of being tipped into instability.

#### Income or rent disruption.

First, I tested whether individuals receiving supplemental security income (SSI) saw a smaller impact on housing instability than those without SSI. Because SSI replaces income for those with disabilities who cannot work, recipients should theoretically not be impacted by income loss. However, even after controlling for admission category (mental health, injury, etc.) and age, SSI recipients did not see a blunted impact of health shocks on housing stability (interaction: −2.6 moves per 1000 people; 95 % CI: −5.6, 0.05).

Second, I tested whether increased length of stay (LOS) was associated with worse impact, hypothesizing that being kept longer would translate to lost days of work and risk of missing rent. Because long stays may simply indicate severity of the condition or unmeasured patient characteristics, I used a proxied version with a “leave-one-out” instrumental variable (or jackknife instrumental variables estimation) (Angrist et al., 1999). Rather than using observed LOS, the proxy calculates the average LOS for all other patients with the same diagnosis at the same hospital, and then uses this average as the “prediction” for each patient’s LOS. This value is then used as the variable in the model. This proxied LOS is theoretically exogenous to the impact of health shocks on housing stability (unlike the patient’s own LOS). This can better isolate variation in hospital-level tendencies to keep patients for more or less time – a policy-relevant characteristic – from a patients’ own severity. The mean LOS at a given hospital for a given diagnosis was highly associated with one’s own LOS, making it a strong instrument (beta = 0.97, 95 % CI: 0.97, 0.98; i.e., a one-day increase in the hospital-level mean LOS is associated with a 0.97-day increase in one’s own LOS). Using this proxied LOS, each additional day kept in a hospital led to 0.7 more moves per 1000 people (95 % CI: 0.5, 0.9) – or, an 8 % increase in effect per additional day.

Finally, I tested if the magnitude of effect varied by whether the person was hospitalized over the first of the month, when most landlords collect rent. Someone admitted on the 29th may be more likely to miss rent or see a breakdown in their landlord relationship than someone admitted on the 5th. After controlling for admission category, age, and LOS, an admission overlapping with the first was not associated with effect size (interaction: 2.5 moves per 1000 people; 95 % CI: −0.1, 5.9).^9^

#### Implicit insurance.

To examine whether “implicit insurance” conferred resilience against a future health shock, I first examined whether subsidized housing was associated with smaller impact sizes. In the face of unstable income or other vulnerabilities, subsidized housing is perhaps one of the strongest protections available, with more eviction oversight and payments that are responsive to fluctuating income (Preston and Reina, 2021). After controlling for admission category, race/ethnicity, and age, having subsidized housing showed substantial protection: the effect was 8.2 moves per 1000 people lower than the effect among market-rate housing tenants (95 % CI: −13.1, −6.5), virtually eliminating any increase in instability after the health shock.

Next, I examined whether having two or more working-aged adults in a household resulted in smaller effects, hypothesizing that a second earner or caregiver might blunt the impact of a health shock. Households with 2+ working-aged adults saw an effect −8.2 moves per 1000 people lower than singles or families with one adult (95 % CI: −11.6, −4.8), suggesting that this form of social support may confer meaningful protection. However, I only observe households sharing a Medicaid case: if someone is on a different case (i.e., financially separate) or not on Medicaid, they are not counted. Given that most Medicaid eligibility is determined at the household level, this metric is likely still picking up a meaningful signal.

Finally, I tested whether having a usual source of outpatient care prior to the shock – or, at least two outpatient visits to the same primary care or outpatient specialist in the two years prior to the shock – was associated with a smaller effect, after controlling for admission category and age. Having a usual source of care resulted in the health shock having a smaller effect on instability, at −8.9 moves per 1000 people lower than those without (95 % CI: −11.5, −6.2).

#### Safety net adequacy.

While certain programs – like subsidized housing and outpatient care – may build resilience against disruptions that future health shocks may bring, it is also possible that quality of the very safety net designed to catch people during a crisis also matters. First, I tested whether quality of inpatient care was associated with instability risk. Using CMS’s star-rating system, I tested whether enrollees admitted to higher-quality hospitals, after controlling for ZIP, age, and diagnosis, experienced a blunted impact of the event on housing instability, hypothesizing that higher-quality care helps patients get on their feet (i.e., home/to work) quickly or connects them with ancillary services necessary for rapid recovery and avoiding instability. Star rating was strongly associated with effect size: each additional star corresponded to a decrease of 1.9 moves per 1000 people in the effect of health shocks (95 % CI: −3.3, −0.6).

Finally, I investigated whether design of the housing safety net mattered by testing whether municipally-owned affordable housing (i.e., NYCHA) was more protective than more diffuse forms of subsidized housing (e.g., section 8 vouchers). While both types of programs provide generous rent subsidies, typically capping rent at 30 % of income or less, municipal housing may offer additional protections against discrimination or eviction due to greater oversight, versus a private Section 8 landlord. Instead, these forms of housing subsidies were equally protective, with both virtually eliminating the relationship between health shocks and mobility (interaction: −0.1 moves per 1000; 95 % CI: −6.9, −6.7).

## 5. Discussion and policy implications

### 5.1. Discussion

This paper provides new evidence disentangling the bidirectional relationship between health and housing, by isolating major health events as one precipitating factor for residential mobility and housing instability, including homelessness. Using high-frequency health data linked to residential addresses among a broad population in a tight urban housing market, I show that adverse health shocks preceded a 21–35 % increase in total quarterly moves, a 40–56 % increase in extreme mobility, and a 6–10 % increase in the risk of going into homelessness (e.g., street, shelter). The effects are even larger when limiting the analyses to urgent admissions. There are approximately 6.5 million non-birth Medicaid hospitalizations annually (Liang et al., 2020). Scaling the baseline housing instability rates and impact estimates from this analysis to the national low-income Medicaid population implies that adverse health events may annually generate around 80,000 additional moves and 10,000–20,000 additional cases of homelessness, in the first quarter after hospitalization alone.

Crucially, these impacts were observed among a well-insured population of low-income New York Medicaid enrollees who face little to no cost-sharing for their care, indicating that the health event itself, beyond financial impacts of medical bills, can trigger cascading social effects like housing instability. Although the settings and methods are not directly comparable across studies, the 20–40 % increase in metrics of housing instability estimated in this analysis is similar to the effect estimated following a felony conviction, job loss, or divorce (Bryan, 2023; Desmond and Gershenson, 2017; Moschion and van Ours, 2019) – and much larger than the impact of neighborhood housing market factors, like gentrification, on residential mobility and instability (Ding et al., 2016; Dragan et al., 2020; Hepburn et al., 2024).

Another way to put these results into context is to use the causal estimate from this analysis to decompose cross-sectional associations between poor health and housing instability. Most prior research has been limited to estimating the cross-sectional association between health events and housing events measured over a given period. For example, the naïve one-year cross-sectional association between hospitalizations and moves in the general New York Medicaid enrollee population is 0.084 (SE: 0.001), or an increase of 0.084 moves per additional hospitalization in a given year. Some portion of this association is due to the impact of poor health on housing instability, some is due to the impact of housing instability on health, and some is due to common third factors that lead to both poor health and housing mobility (e.g., divorce). Using the causal estimates in this paper, we can calculate that about 40–50 % of the total association is due to the *impact of poor health on housing outcomes*.^10^ Offering evidence that a large share of the housing-health correlation may be due to “reverse causality” is an important contribution, as nearly all research and policy recommendations in this area interpret the correlation between poor health and housing mobility and instability as only reflecting housing as a social determinant of health, rather than as housing outcomes as a *consequence* of poor health.

Heterogeneity analyses show that sizeable increases in mobility occurred across all types of hospitalizations, except non-urgent admissions like joint replacements (“placebo” shocks). This is an important contribution, as prior work has only focused on single types of shock (e.g., traffic injuries or children born with congenital conditions). Another striking finding is the similarity in effect sizes for children and adults. Prior work has documented the spillover of health events to the wellbeing of other members of a family (Breivik and Costa-Ramón, 2021; García-Gómez et al., 2013). The fact that health shocks to any member of a family generate a similar increase in moves for the household points to the importance of considering the entire family unit when assessing the tail of social and economic consequences from health events.

This paper also sought to isolate policy-relevant characteristics that were driving variation in the effect size. In these analyses, I found a smaller magnitude for those with subsidized housing, shorter lengths of stay, higher quality of care, greater social support, and consistent outpatient relationships. These policy analyses, which are explored more below, can inform future trials of pilot interventions.

For income or rent related mechanisms, I found surprisingly little evidence, with neither SSI nor admission over the 1st of the month (i.e., rent due date) substantially altering the effect size. It is possible that income and rent disruptions are simply not as salient of mechanisms as theorized. However, there are a few more nuanced potential explanations. First, the Social Security Administration limits payments to beneficiaries during hospitalizations by default, unless specifically contacted to request continuation (Social Security Administration, 2023). Also, SSI recipients are disabled, and may face discrimination by landlords or experience other hardships following a health decline, counteracting protective effects (Hammel et al., 2017; Jarwala and Singh, 2019; Levy et al., 2015). Finally, family members who may supplement housing costs for the SSI recipient may have to reduce work themselves to support the hospitalized enrollee, resulting in income-related disruptions anyway. Likewise, we might not see variation in the effect by the day of the month if low-income individuals are not always on 1st-of-the-month schedules (week-to-week leases or motels). There may be too much heterogeneity in people’s work and rental situations to detect clear evidence of this pathway, without more precise data on employment, wages, and rent.

I do observe substantial variation in the effect by the amount of time a patient is kept in the hospital, even after using a leave-one-out proxied measure that isolates the plausibly exogenous portion of one’s length of stay. Being kept in a hospital longer may disrupt individuals’ lives more permanently, whether that is through missing excessive days of work, disruptions to their family’s lives, or other pathways. Low-wage workers and precariously employed people are known to have fewer protections against employment dismissal, including less awareness of policies like paid leave (Rossin-Slater, 2018).

Enhancing access to forms of “implicit insurance” and other supports prior to a shock may be highly protective. Subsidized housing conferred one of the most powerful protective effects, virtually eliminating the relationship between health problems and instability. This is consistent with other research showing the protection subsidized housing confers against stressors (Dragan et al., 2020, 2019). Likewise, the presence of two working-aged adults conferred protection, as did having a well-established, regular outpatient relationship prior to the shock. Regular outpatient care in particular may establish trust and familiarity in the social service system: during crises, patients have experience navigating assistance or treatments and can readily be linked to other services (Starfield et al., 2005). This set of findings may, in part, be simply picking up selection of “better off” patients who may be less likely to experience instability after a health event anyway (e.g., more health-conscious, savvier at navigating services). Still, these protective effects suggest there may be ways to buoy families at risk of health crises.

Finally, the quality of the health care safety net is important. Low-quality care during the shock was strongly linked to the magnitude: patients going to higher-quality hospitals were far less likely to experience subsequent housing mobility, even after accounting for patient characteristics and severity. While research has documented inequities in clinical outcomes stemming from low-quality or inadequate care (Chandra et al., 2023), this suggests that low-quality care may have consequences for other social outcomes, like housing, as well. More fundamentally, this study design brings into relief how health shocks disrupt a general trend toward increased housing stability for long-term Medicaid enrollees.

The high-frequency, individual-level data used in this paper provide novel insights into the complex relationship between housing and health. However, it has limitations. First, this paper focuses on the New York City context, which may not generalize to the broader US. Still, a large share of people experiencing homelessness live in NYC and similar cities like Los Angeles, San Francisco, Seattle, Boston, Chicago, and Washington D.C., with NYC accounting for 1 in 5 homeless individuals in the US alone (Office of the New York State Comptroller, 2025). Additionally, it limits the analyses to people consistently on Medicaid, which may represent a more stable and well-connected group of low-income New Yorkers. The literature lacks consistent and validated metrics of housing instability, largely due to data limitations; the metrics used in this analysis rely on measures from prior research when possible (Kang, 2021; Pourat et al., 2023; Vickery et al., 2018; Zech et al., 2015), but may not fully capture the constructs of housing instability and homelessness that they intend to. This is especially true for the “extreme mobility” measure, which has not been used in prior literature. Likewise, it is difficulty to know for certain whether a sudden hospitalization after two hospital-free years is a true adverse event or “shock”; however, using this definition aligns with prior work (Dobkin et al., 2018), and this approach excludes people with increasing health system utilization in the quarters leading up to the shock, to eliminate anticipated admissions.

### 5.2. Policy implications

In the short run, understanding health events as tipping points into residential instability for precariously-housed populations in unforgiving housing markets is important for the design of rapid cross-sector preventive efforts. While insufficient affordable housing and high housing costs are theorized to be as the primary driver of residential instability in the United States, (Bailey, 2022; Colburn and Aldern, 2022; Quigley and Raphael, 2001; Shinn and Khadduri, 2020), large-scale housing reform takes massive political will, resources, and time. Designing protections for people on the precipice of instability, like those experiencing poor health, is a key complement to these goals.

This work contributes to the debate around health systems’ roles in non-health domains. Health organizations are becoming interested in interventions to break the housing-health cycle, yet many such initiatives have been constrained by regulations about what health dollars can be used for (Courtin et al., 2020; Horwitz et al., 2020). While some argue that health systems and insurers should become more active in building housing or offering reimbursement of housing costs, others worry about the consequences of health systems becoming housing providers themselves, including the misdirection of dollars that could be used by the housing sector (Butler, 2022; Glied and D’Aunno, 2023; Gondi et al., 2020). This paper approaches this issue from a different angle: it offers evidence for how health systems might use their existing skills and comparative advantage to support patients on the precipice of instability, by protecting them from disruptive health events in the first place – or offering higher-quality care to limit the long tail of social consequences when they do fall ill. Adverse health events are a tipping point into mobility and instability particularly amenable to health system action, yet nearly all research on the housing-health relationship has focused only on the role of housing in shaping health. Pilot programs to understand policy or programmatic options for breaking this relationship could include medical-legal partnerships during inpatient stays that can improve patients’ access to legal advocates to help with both housing (eviction) and health (quality of care) issues; assistance applying for paid leave, subsidized housing, emergency rent assistance, or disability accommodations prior to discharge; or interventions to improve access to outpatient care, such as community health workers. More broadly, it is possible that improving quality of care for low-income people generally – e.g., through improved access to gold-standard care like novel depression treatments, highly effective medications for diabetes, HIV, and hepatitis, buprenorphine for opioid use disorder, and preventive services like PrEP – might protect marginal patients from falling into poor housing outcomes by simply reducing risk of health crises in the first place.

Given longstanding evidence of inequitable health outcomes for low-income populations, these findings highlight the need to reduce those inequities, for reasons beyond clinical outcomes. Further, by focusing on well-insured Medicaid enrollees who do not face cost-sharing for care, this paper highlights that insurance alone is not the full story when protecting people from the long tail of consequences from adverse events. People experiencing illness are uniquely vulnerable to residential mobility and housing instability, and reducing this risk may be a complementary objective of policies addressing access to affordable housing.

## Figures and Tables

**Table 1:** Demographic characteristics.

	Experienced a Health Shock			No Shock
	Overall	Children	Adults	Comparators
N	(3–64) 237,199	(3–17) 46,207	(18–64) 190,992	(3–64) 237,199
Race/ethnicity (%)				
Asian	7.5	6.5	7.7	7.5
Black	27.5	24.5	28.2	27.5
Hispanic	30.1	31.0	29.9	30.1
White	14.2	9.5	15.4	14.2
Other	5.8	3.5	6.3	5.8
Unknown	15.0	25.0	12.6	15.0
Female (%)	56.5	44.8	59.3	56.5
Age (median, IQR)	42.1 (23.0–53.8)	10.3 (6.1–14.6)	47.6 (34.8–55.6)	41.9 (22.5–53.7)
Top admission reasons (%)				
	Mood (5)	Asth (14)	Schiz (5)	
	Asth (5)	Mood (9)	Mood (4)	
	Schiz (4)	Append (8)	Subst (4)	
	Skin (3)	Epilep (5)	Bariat (3)	
	Subst (3)	Pneum (5)	Septic (3)	

*Notes:* Abbreviations for admission reasons correspond to the following CCS categories: Mood>Mood Disorders; Asth>Asthma; Schiz>Schizophrenia and psychotic disorders; Bariat>Nutritional and metabolic disorders (empirically, this is mostly bariatric surgery admissions); Skin>Skin and tissue infections; Append>Appendicitis; Epilep>Epilepsy and convulsions; Pneum>Pneumonia; Subst>Substance use disorder; Septic>Septicemia.

*Source:* Author calculations of New York State Medicaid claims and encounters data.

**Table 2:** Main effect of the health shock on housing outcomes after the health shock at the first quarter after the shock and the fifth quarter after the shock.

	Total moves^a^	Extreme mobility^b^	Homelessness^c^	Housing quality^d^	Tract pov^e^	Distance^f^
**Effect at first q. (95 % CI)**	12.2*** (10.3, 14.0)	0.2*** (0.1, 0.2)	0.3*** (0.1, 0.5)	0.0 (−1.1, 1.1)	−0.01 (−0.1, 0.1)	0.1 (−0.2, 0.4)
Expected mean (counterfactual)	48.8	0.40	5.3	30.4	28.4	4.5
Relative change	25 %	45 %	6 %	0 %	0 %	2 %
**Effect at fifth q. (95 % CI)**	6.7*** (4.8, 8.5)	0.1*** (0.1, 0.2)	0.5*** (0.3, 0.7)	0.1 (−1.0, 1.2)	0.01 (−1.1, 1.2)	0.4** (0.1, 0.7)
Expected mean (counterfactual)	46.6	0.38	5.0	30.4	28.4	4.2
Relative change	14 %	29 %	10 %	0 %	0 %	9 %
N	474,398	474,398	474,398	304,738	424,952	58,474

*Notes:* Maintenance violations data was only available 2014–2019. All other data spans the entire period from 2010–2019. This table shows the immediate effect of the health shock on each outcome as explained in Eq. (1) in the methods. The relative change is calculated as the percent increase or decrease from the predicted value of the outcome in the absence of a shock (i.e., based on the extrapolated pre-period difference).

*Source:* Author calculations of New York State Medicaid claims and encounters data and Department of Housing Preservation and Development Maintenance Violations Data.

a Rate per 1000 people per quarter.

b Percent. Change is expressed in percentage point increases or decreases. This refers to a person who reports two or more unique addresses per quarter.

c Percent. Change is expressed in percentage point increases or decreases. This refers to people likely experiencing homelessness or the state of living on the street, in a known shelter, in a jail without another address, or reporting the headquarters of a known social service organization as their address.

d Building-level maintenance violations per 100 units.

e Percent of residents in census tract living in poverty per the US Census. If the enrollee lived in more than one tract in the quarter, the average poverty rate was taken. Moves to shelters or using the addresses of social service organizations were excluded from this analysis.

f Distance moved in miles, conditional on moving. Only quarters containing a move and addresses in NYC were included in this analysis.

*** Significant at the 1 percent level.

** Significant at the 5 percent level.

* Significant at the 10 percent level.

**Table 3:** Sensitivity Analysis and Alternative Outcomes.

	Total moves^a^		Extreme mobility^b^		Homelessness^c^		Housing quality^d^	
	Effect (CI)	Rel. Change	Effect (CI)	Rel. Change	Effect (CI)	Rel. Change	Effect (CI)	Rel. Change
**Panel A: Sensitivity analyses**								
Remove non-urgent procedures (*n* = 256,308)	19.0*** (16.3, 21.6)	35 %	0.3*** (0.2, 0.4)	56 %	0.5*** (0.2, 0.8)	7 %	−0.1 (−1.7, 1.5)	0 %
Ambulance arrivals only (*n* = 82,620)	10.7*** (7.4, 13.9)	23 %	0.2*** (0.1, 0.2)	43 %	0.4*** (0.1, 0.8)	7 %	0.1 (−2.0, 2.2)	0 %
Control for quarterly healthcare visits (*n* = 474,398)	11.5*** (9.7, 13.4)	24 %	0.1*** (0.1, 0.2)	40 %	0.3*** (0.1, 0.5)	6 %	0.2 (−0.9, 1.3)	1 %
**Panel B: Alternative metrics of housing instability**								
Housing instability diagnosis codes per 100 people (*n* = 240,982)	12.5*** (10.1, 15.0)	269 %						
Remove moves to/from health facilities (*n* = 474,398)	10.1*** (8.3, 12.0)	21 %						

*Notes:* The estimates in this table come from the event study model described in Eq. (1) and capture the effect at quarter 0 relative to the pre-event difference with the comparator group. Maintenance violations data was only available 2014–2019 and housing instability diagnostic codes were available 2015–2019 only. All other data spans the entire period from 2010–2019. The relative change is calculated as the percent increase or decrease from the predicted value of the outcome in the absence of a shock.

*Source:* Author calculations of New York State Medicaid claims and encounters data and Department of Housing Preservation and Development Maintenance Violations Data.

a Rate per 1000 people per quarter.

b Percent. Change is expressed in percentage point increases or decreases. This refers to a person who reports two or more unique addresses per quarter.

c Percent. Change is expressed in percentage point increases or decreases. This refers to people likely experiencing homelessness, or the state of living on the street, in a known shelter, in a jail without another address, or reporting the headquarters of a known social service organization as their address.

d Building-level maintenance violations per 100 units

*** Significant at the 1 percent level.

** Significant at the 5 percent level.

* Significant at the 10 percent level.

## Data Availability

The data that has been used is confidential.

## Footnotes

1 Among adults, study designs have largely only been associational and cross-sectional. Houle and Keene (2015), Leonard, Hughes, and Pruitt (2017), and Nilsson and colleagues (2024) found associations between worsening health and instability, but could not isolate temporality well due to the nature of their data.

2 For more discussion of the high prevalence of and mechanics of informal evictions and other forced moves, see Desmond (2016).

3 The exact procedure for producing the pool of potential matches is as follows: (1) All enrollees with a 2+ year period without a hospitalization were identified; (2) A random date within that period was assigned, such that at least 2 years of hospital-free time and continuous enrollment remained in the pre-period, and 2 years of continuous enrollment remained in the post-period; and (3) enrollees from this pool were exact matched to enrollees with a health shock (see match variables in text).

4 All but 4% of enrollees were matched to a comparator. The 4% of unmatched enrollees were not substantially demographically different from the matched enrollees, but they were slightly more likely to be male (49% in unmatched vs 43% in matched) and White (18% in unmatched vs 14% in matched) and slightly less likely to be Hispanic (21% in unmatched vs 29% in matched).

5 Results aggregating moves by month since hospitalization (rather than quarter/3-month windows) are available in the Appendix (A3).

6 Data on housing violations were only available 2014–19 from the NYC Department of Housing Preservation and Development’s OpenData portal. Thus, housing quality analyses are limited to these years.

7 Because these codes only exist from 2015 onward, I only run this sensitivity analysis for health shocks occurring in 2017 to afford two years of data before and after the shock.

8 Disenrollment may be associated with more residential mobility (e.g., people whose lives are so dramatically disrupted by the health event that they fall out of the safety net or leave New York altogether) or less residential mobility (if they sought and secured a higher paying job with employer sponsored insurance to cope with the change in health status).

9 Note that in the analysis, stays longer than 28 days were excluded.

10 I thank an anonymous reviewer for the suggestion of contextualizing the findings in this way. The logic driving this back-of-the-envelope calculation is as follows: the naïve cross-sectional association between hospitalizations and residential moves using the New York Medicaid data is 0.0841, off of a baseline move rate of 0.1716 moves per year (for those with 0 hospitalizations), or a 49% increase in moves for each additional hospitalization. If the entire association were due to the effects of health shocks on mobility as estimated in this paper, we should expect a 20–25% increase in moves for those with a hospitalization, or 0.2059 to 0.2145 moves total. Because we see actually 0.2557 moves for those with 1 hospitalization (0.1716+0.0841), we can decompose the total correlation into the expected increase as estimated from this paper versus the total correlation, or (0.2059–0.1716)/0.0841 = 41%, or 51% with the 25% effect estimate. Thus, 40–50% of the total hospitalization-housing instability association is attributable to the effect of health events on housing instability. This leaves the remaining 50–60% of the health-housing to be due to a combination of the impacts of housing instability on poor health and third common factors like divorce.

## Appendix

**Appendix Table A1:** Mapping of CCS categories to analytic groupings.

Category	CCS Category	CCS Label
Mental Health	650	Adjustment disorders
	651	Anxiety disorders
	652	Attention-deficit conduct and disruptive behavior disorders
	653	Delirium dementia and amnestic and other cognitive disorders
	654	Developmental disorders
	655	Disorders usually diagnosed in infancy childhood or adolescence
	656	Impulse control disorders NEC
	657	Mood disorders
	658	Personality disorders
	659	Schizophrenia and other psychotic disorders
	662	Suicide and intentional self-inflicted injury
	670	Miscellaneous disorders
Substance Use Disorders	660	Alcohol-related disorders
	661	Substance-related disorders
Injury	225	Joint disorders and dislocations; trauma-related
	226	Fracture of neck of femur (hip)
	227	Spinal cord injury
	228	Skull and face fractures
	229	Fracture of upper limb
	230	Fracture of lower limb
	231	Other fractures
	232	Sprains and strains
	233	Intracranial injury
	234	Crushing injury or internal injury
	235	Open wounds of head; neck; and trunk
	236	Open wounds of extremities
	239	Superficial injury; contusion
	240	Burns
	244	Other injuries and conditions due to external causes
Cardiac Event or Stroke	96	Heart valve disorders
	97	Peri-; endo-; and myocarditis; cardiomyopathy
	98	Essential hypertension
	99	Hypertension with complications and secondary hypertension
	100	Acute myocardial infarction
	101	Coronary atherosclerosis and other heart disease
	102	Nonspecific chest pain
	103	Pulmonary heart disease
	104	Other and ill-defined heart disease
	105	Conduction disorders
	106	Cardiac dysrhythmias
	107	Cardiac arrest and ventricular fibrillation
	108	Congestive heart failure; nonhypertensive
	109	Acute cerebrovascular disease
	110	Occlusion or stenosis of precerebral arteries
	111	Other and ill-defined cerebrovascular disease
	112	Transient cerebral ischemia
	113	Late effects of cerebrovascular disease
	114	Peripheral and visceral atherosclerosis
	117	Other circulatory disease
Other Urgent Diagnoses	2	Septicemia
	5	HIV infection (including complications)
	49	Diabetes mellitus without complication
	50	Diabetes mellitus with complications
	51	Other endocrine disorders
	61	Sickle cell anemia
	76	Meningitis
	77	Encephalitis
	83	Epilepsy; convulsions
	115	Aortic; peripheral; and visceral artery aneurysms
	116	Aortic and peripheral arterial embolism or thrombosis
	122	Pneumonia
	127	Chronic obstructive pulmonary disease and bronchiectasis
	128	Asthma
	130	Pleurisy; pneumothorax; pulmonary collapse
	131	Respiratory failure; insufficiency; arrest (adult)
	142	Appendicitis and other appendiceal conditions
	153	Gastrointestinal hemorrhage
	157	Acute renal failure
